# An investigation of Ca-doped MgO nanoparticles for the improved catalytic degradation of thiamethoxam pesticide subjected to visible light irradiation

**DOI:** 10.1038/s41598-024-51738-9

**Published:** 2024-01-11

**Authors:** Huma Khalid, Atta ul Haq, Ameer Fawad Zahoor, Ali Irfan, Magdi E. A. Zaki

**Affiliations:** 1 Department of Chemistry, Government College University Faisalabad, Faisalabad, Pakistan; 2 Department of Chemistry, College of Science, Imam Mohammad Ibn Saud Islamic University (IMSIU), 13623 Riyadh, Saudi Arabia

**Keywords:** Environmental sciences, Chemistry, Materials science, Nanoscience and technology

## Abstract

The remediation of pesticides from the environment is one of the most important technology nowadays. Herein, magnesium oxide (MgO) nanoparticles and calcium-doped magnesium oxide (Ca-doped MgO) nanoparticles were synthesized by the co-precipitation method and were used for the degradation of thiamethoxam pesticide in aqueous media. Characterization of the MgO and Ca-doped MgO nanoparticles were performed by XRD, SEM, EDX, and FT-IR analysis to verify the synthesis and variations in chemical composition. The band gap energy and crystalline size of MgO and Ca-doped MgO nanoparticles were found to be 4.8 and 4.7 eV and 33 and 34 nm respectively. The degradation of thiamethoxam was accomplished regarding the impact of catalyst dosage, contact time, temperature, pH, and initial pesticide concentration. The pH study indicates that degradation of thiamethoxam depends on pH and maximum degradation (66%) was obtained at pH 5 using MgO nanoparticles. In contrast, maximum degradation (80%) of thiamethoxam was observed at pH 8 employing Ca-doped MgO nanoparticles. The percentage degradation of thiamethoxam was initially increasing but decreased at higher doses of the catalysts. The degradation of the pesticide was observed to be increased with an increase in contact time while high at room temperature but decreased with a temperature rise. The effect of the initial concertation of pesticide indicates that degradation of pesticide increases at low concentrations but declines at higher concentrations. This research study reveals that doping of MgO nanoparticles with calcium enhanced the degradation of thiamethoxam pesticide in aqueous media.

## Introduction

The majority of the nation’s economy relies heavily on agriculture and helps the country’s population directly. Cotton, rice, fruits, vegetables, and wheat are the major crops. The irrigation system is one of the biggest system to support the agriculture. Utilizing resources more effectively, particularly land and water, is the principal requirement for agricultural production^1^. The land, water, agronomic, climate, and socioeconomic challenges facing the agriculture sector have significant effects on agricultural productivity. To enhance agricultural productivity various strategies are required to improve water and non-water management of agricultural productivity^2^. The attack of pests is one of the major issues for crop production. Pest infestations account for approximately 45% of annual food production losses: thus, efficient pest management with pesticides is compulsory to combat pests and boost crop production^3^. It can be removed by using varieties of pesticides because pesticide provides numerous agricultural benefits. The application of pesticides in forestry, public health, and agriculture has resulted in tremendous benefits. High-yield seed varieties, agricultural chemicals, and cutting-edge irrigation techniques all contributed to this outcome^4^. All over the world, pesticides are widely employed to control insects, pests, and various plants diseases but there is some concern about environmental safety^5^.

The first commercial neonicotinoid insecticide in the thionyl subclass is thiamethoxam^6^. The thiamethoxam has demonstrated enormous benefits in industries, domestic landscapes, forests, and agriculture^7^. Various biting and sucking insects like white flies, thrips, and aphids are effectively prevented by this insecticide. It has a wide range of certain physical and chemical properties such as seed treatment, soil drench foliar and seed applications. Corn seed has been treated with this insecticide. Neonicotinoid is effective against pest insects that are resistant to other insecticide classes like chlorinated hydrocarbons, carbamates, organophosphates, and pyrethroids^8^. However, the evidence that is currently available indicates that insecticides may pose a risk to aquatic invertebrates, humans, honey bees, and non-target insects. But the excessive use creates a serious threat to aquatic life and also raises environmental risks^7^.

Pesticide contamination of water typically results from agricultural run-off and toxin-producing wastewater^9^. The most pressing issue is their impact on natural and human health^10^. Due to the toxic effects of thiamethoxam on human beings and the ecosystem, this chemical substance must remove from soil and water sources using efficient and environmentally safe methods^11^.

Numerous approaches can be taken like flocculation, bioremediation, coagulation, ozonation, photo-Fenton, and photocatalysis for the degradation and removal of this contaminant^12,13^. These methods have some limitations, such as operational difficulty, formation of waste products, and high cost^14^.

In recent years, photocatalysis has been one of the advanced oxidation processes that has paid substantial immersion because of low power consumption, using cheap catalysts, undergoing complete degradation of contaminants, and its simplicity in operating^15^. In this method, a catalyst, radiation, and oxidizing agents combine to convert organic material into less hazardous inorganic compounds. This method produces hydroxyl radicals with low selectivity and high oxidative power. Resultantly, numerous toxic compounds are transformed into non-toxic and highly degradable substances. In the photocatalytic process, catalysts are used which are low-cost, nontoxic, and extremely stable substances^16^. During the last decade, novel materials have been developed and evaluated for the decontamination of wastewater containing toxic and persistent pollutants^17–21^.

The magnesium oxide is widely accepted due to its high stability, non-toxicity, environmentally friendly having a large band gap of 7.8 eV, low refractive index, and dielectric constant1^22,23^. The magnesium oxide nanoparticles provide a large surface area to enhance the rate of thiamethoxam adsorption^24^.

During the last decade, many researchers focused their attention on enhancing the photocatalytic efficiency of the photocatalysts by decreasing the recombination of electron–hole pairs, decreasing the band gaps, and extending the absorption ability of the photocatalyst in the visible region because most of the solar radiation is composed of visible radiation^25^. In the recent past, the researchers have focus their attention to improve the catalytic activities of the photocatalysts by doping of an appropriate atom in the crystalline structure of photocatalysts^26–33^. The fact that MgO semiconductor uses a small range of UV light is the primary drawback of photocatalysis. This issue can be resolved by taking a slight modification of MgO nanoparticles to widen the band gap by doping other heterometal atoms. Therefore, calcium was used as a dopant to hinder the fast recombination of electron–hole pairs and provide a wide band gap range^34^.

Therefore, in the current research, MgO nanoparticles and Ca-doped MgO nanoparticles were synthesized through co-precipitation method. The MgO and Ca-doped nanoparticles were then employed for the degradation of thiamethoxam in aqueous media concerning the influence of the dosage of catalyst, contact time, temperature, pH of the solution, and initial pesticide concentration. Moreover, the stability and reusability of MgO nanoparticles and Ca-doped MgO nanoparticles were also studied in the current research work.

## Experimental

### Materials

The analytical pure chemicals of calcium chloride, magnesium sulfate, boric acid, phosphoric acid, acetic acid and thiamethoxam were purchased from Sigma-Aldrich Germany and Merck chemical company Germany, and were used without any further purification.

### Preparation of standard solution of thiamethoxam

To prepare a stock solution of 1000 µgmL^−1^ of thiamethoxam, an accurately weigh quantity of 0.25 g was transferred in a beaker (250 mL) and dissolved in an appropriate quantity of distilled water. The beaker content was then transferred in to a volumetric flask of capacity of 100 mL and diluted with distilled water up to mark. Dilute solutions of thiamethoxam of known concentrations in 100 mL volumetric flasks were prepared by diluting the stock solution for further studies.

### Preparation of MgO nanoparticles and Ca-doped MgO nanoparticles

In this research work, MgO and Ca-doped MgO nanoparticles were synthesized through co-precipitation method. Firstly, accurately 7.84 g of KOH (140 mmol) was dissolved in a beaker of 250 mL capacity and transferred in 100 mL volumetric flask and, then diluted up to marks with distilled water. This solution was marked as solution A. In the second place, an accurately weigh quantity; 2.4 g of magnesium sulfate (20.1 mmol) was dissolved in a beaker with a sufficient quantity of water. The solution was then transferred in a 100 mL volumetric flask and diluted with distilled water up to mark. This solution was marked as solution B. Similarly, a solution of MgO doped with calcium was prepared by dissolving 2.4 g of magnesium sulfate (20.1 mmol) and 0.2 g of calcium chloride (2.4 mmol) in a beaker with a sufficient amount of distilled water and transferred in 100 mL volumetric flask. The solution was finally diluted up to marks with distilled water and assigned as solution C. After preparation of these solutions, solutions B and C were heated at 52 °C and KOH solution was added dropwise in both solutions with constant stirring and was refluxed for 2 h. The white precipitate of MgO nanoparticles and Ca-doped MgO nanoparticles were obtained. These precipitates were washed with distilled water and allowed to cool at room temperature. The resultant products were dried in the air to attain the high crystalline quality of nanoparticles. A similar procedure has followed in the literature for the synthesis of ZnO and Co doped ZnO nanoparticles^35^.

### Characterization techniques

Characterization of the prepared catalysts was done using various techniques. The SEM technique (SEM-Model-JSM-5910, Japan JEOL) was used for the morphological and structural study of MgO nanoparticles and Ca-doped MgO nanoparticles while EDX technique (EDX-INCA 200 Oxford Instruments UK) was employed for elemental compositional of the MgO nanoparticles and Ca-doped MgO nanoparticles. The FTIR (Mechelle 5000) and XRD (JDX-3532 JEOL, Japan) techniques were used for the determination of functional groups and crystallography of MgO nanoparticles and Ca-doped MgO nanoparticles respectively. The UV–Vis double beam spectrophotometer (C-7200S, Peak Instruments Ins. USA) was utilized for the determination of concentration thiamethoxam pesticide in the aqueous media.

### Degradation experiment

The degradation of thiamethoxam was determined using MgO nanoparticles and Ca-doped MgO nanoparticles as catalysts in the presence of sun light as a source of visible radiation. The degradation of thiamethoxam was carried out by taking 10 mL of pesticide having concentration ranged of 2–20 µgmL^−1^ regarding the influences of different experimental operational parameters such as initial pH, catalytic amount, temperature, contact time, and initial thiamethoxam concentration. In this experiment, the catalyst dose lies in the range of 0.01–0.1 g by adjusting the solution pH from 3 to 12 at different temperatures in the range of 30–80 °C. The concentration of thiamethoxam pesticide after degradation was investigated at 240 nm by UV/Visible spectrophotometer. The percent degradation of thiamethoxam was evaluated by following equation:

$$Degradation \left( \% \right) = \left[ {\frac{{C_{o} - C_{t} }}{{C_{o} }}} \right] \times 100$$

Herein, C_o_ represents the initial concentration of thiamethoxam and C_t_ designates the concentration of thiamethoxam after the desire time interval.

## Results and discussion

### Effect of pH

**Figure 1:**
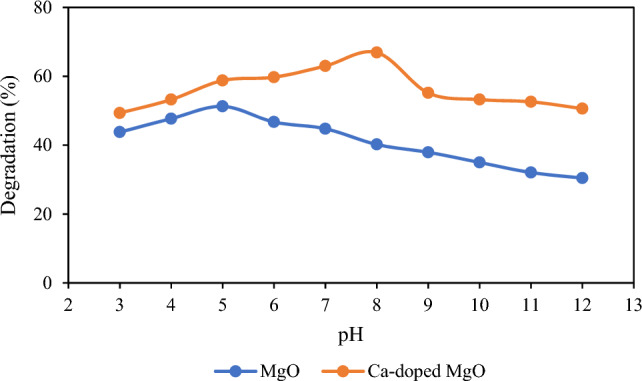
Effect of pH on the degradation of thiamethoxam.

According to reports published the solution pH is an important parameter which significantly influences the rate of catalytic reaction and the chemistry of the solution. Hence, to investigate and observed the influence of solution pH on the photocatalytic degradation of thiamethoxam pesticide, various experiments were performed by changing the pH in the range of 3–12 while other experimental parameters were kept constant. The result is shown in Fig. 1 which indicates that MgO nanoparticles show maximum degradation of thiamethoxam at 5.0 pH but thiamethoxam pesticide was degraded maximum at pH 8.0 when Ca-doped MgO nanoparticles was used as photocatalyst. However, the degradation of thiamethoxam was enhanced in the case of Ca-doped MgO nanoparticles as compared with MgO nanoparticles.

It may be explained that surface of the catalyst attains more positive charges at pH 5.0 resulting the facilitation of adsorption of more negatively charged thiamethoxam molecules which subsequently leads maximum degradation. However, below pH 5.0, the number of hydrogen ions was increased and results the capturing of photogenerated electrons which lead to lowering of the photocatalytic degradation of thiamethoxam. When the pH of the solution lies in the basic medium the degradation rate decreases because the surface of the catalyst contains more negative charges which undergo electrostatic repulsion between the catalyst surface and the pesticides molecule in the solution which leads to the decrease in degradation^36^. Consequently, further degradation of thiamethoxam was studied at pH 5 and pH 8 using MgO nanoparticles and Ca-doped MgO nanoparticles respectively.

### Effect of catalytic dose

**Figure 2:**
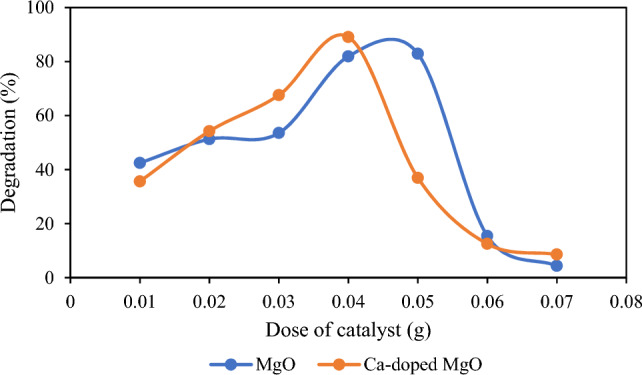
Effect of catalyst dose on the degradation of thiamethoxam.

In the photocatalysis experiments, the optimized amount of prepared catalyst is necessary to prevent the excessive use of photocatalysts. This optimization study was done by accomplishment of various experiments in which catalytic dose was varied from 0.01 to 0.06 g while keeping other factors remains unchanged. The result is shown in Fig. 2 which indicates that photocatalytic degradation of thiamethoxam was increased with the augmentation of catalyst dose from 0.01 to 0.05 g of MgO nanoparticles and 0.01–0.04 g of Ca-doped MgO nanoparticles. However, after these doses of the catalysts, degradation of thiamethoxam started to decrease with further augmentation in catalytic dose. Hence, the maximum degradation of thiamethoxam was found at 0.05 g MgO nanoparticles whereas photodegradation of thiamethoxam was maximum at 0.04 g by Ca-doped MgO nanoparticles. The reason is that more active sites are available with the increase of catalytic dose which in turn increases the degradation efficiency^37^. A reduction in the degradation of thiamethoxam was observed as the dose of catalyst was increased after optimum dose. This may occur due to the agglomeration of the nanoparticles in the solution and turbidity of the solution by high doses of the nanoparticles which prevents penetration of radiation essential for the activation of catalytic surface. Therefore, further degradation process of thiamethoxam was executed at these optimized catalyst doses^38^.

### Effect of time

**Figure 3:**
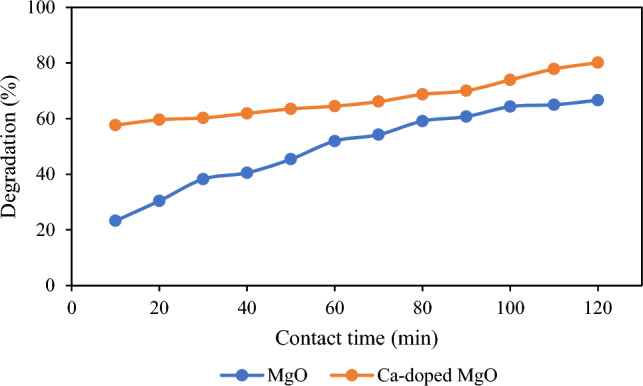
Effect of time on the degradation of thiamethoxam.

It has been studied that time of contact plays a significant and key character in photocatalytic degradation. Hence, the influence of contact time on the photocatalytic degradation of thiamethoxam was studied by varying the contact time from 10 to 120 min while all the other experimental variables were kept without change. The outcome is illustrated in Fig. 3. It has been observed that the degradation of thiamethoxam increases with the time of contact by using MgO nanoparticles and Ca-doped MgO nanoparticles as catalysts^16^. The photocatalytic efficiency of thiamethoxam was enhanced from 23 to 66% using MgO nanoparticles and 57 to 80% using Ca-doped MgO nanoparticles with an augmentation of contact time from 10 to 120 min. On the surface of MgO nanoparticles and Ca-doped MgO nanoparticles as the catalyst, where hydroxyl radicals are entrapped in the reactive species holes, photocatalytic degradation of thiamethoxam takes place. The bonds in the pesticide molecules that are adsorbed on the catalyst surface can be broken down by the hydroxyl radical. The pesticide concentration and the catalyst dose remain constant, but hydroxyl radicals increase as the contact time was increased, and the pesticide molecules are completely broken down into smaller ones^39^.

### Effect of initial concentration of thiamethoxam

**Figure 4:**
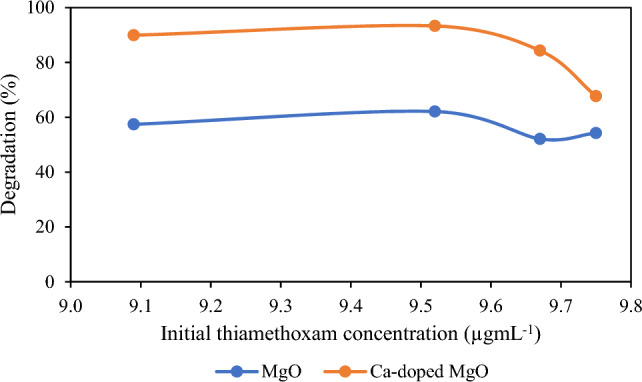
Effect of initial pesticide concentration on the degradation of thiamethoxam.

Attention was drawn that the initial concentration of pesticides has played a major function in the degradation of pesticides. Hence, initial thiamethoxam concentration was changed from 9 to 10 µg mL^−1^ while other parameters remained unchanged. The result of this parameter is shown in Fig. 4 which illustrates that the degradation of thiamethoxam pesticide was enhanced with an increase in the concentration of thiamethoxam pesticide but a decrease was observed at higher initial concentration. The reason is that the number of hydroxyl radicals is insufficient on the surface of the catalyst and the pesticide molecule completely covered the active sites on the surface of catalyst at a low initial pesticide concentration^40,41^. Because hydroxyl radicals are very important for photocatalytic degradation However, at higher initial concentrations, the degradation of thiamethoxam declined owing to the reduction in the number of active sites available for pesticide molecules. Another reason is that more photons are absorbed by the concentration of pesticide molecules, which, in turn, reduces the number of photons that can be used in the photocatalytic reaction^42^.

### Effect of temperature

**Figure 5:**
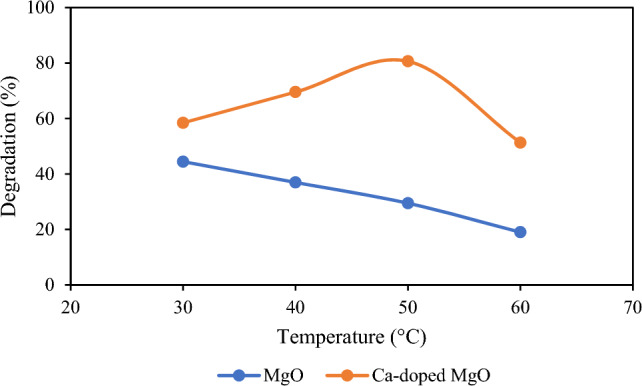
Effect of temperature on the degradation of thiamethoxam.

The temperature of the system has a pronounced influence on the rate of photocatalytic degradation of pollutants. Therefore, the impact of temperature on the photocatalytic degradation of thiamethoxam was scrutinized by changing the temperature from 30 to 60 °C while other experimental parameters were remaining unchanged. The result is shown in Fig. 5 which indicates that degradation of thiamethoxam was enhanced with the rise in temperature by Ca-doped MgO nanoparticles up to a certain level but decreased continuously in the case of MgO nanoparticles. However, at high-temperature degradation efficiency was declined using MgO nanoparticles as well as Ca-doped MgO nanoparticles^43^.

In the course of this study, the thiamethoxam are adsorbed on the surface of Ca-doped MgO nanoparticles and the degradation rate enhances as the temperature rises. The reason is that at higher temperatures the kinetic energy increases which increases the mobility of pesticide molecules to the surface of MgO nanoparticles, and Ca-doped nanoparticles. As a result, more interaction takes place between pesticide molecules and the catalyst which enhanced the degradation process. However, after certain higher temperature levels, the degradation of thiamethoxam started decreasing. It may be suggested that at high temperatures a decrease in the adsorptive force between active sites of the catalysts and pesticide molecules occurs which leads to low degradation at elevated temperatures. On the other hand, using MgO nanoparticles the degradation rate of thiamethoxam constantly decreases with the rise in temperature. It may occur due to the increases in the fast recombination rate of holes and electrons which is responsible for the desorption of thiamethoxam molecules on the surface of MgO nanoparticles^13^.

### Characterization

#### SEM analysis

**Figure 6:**
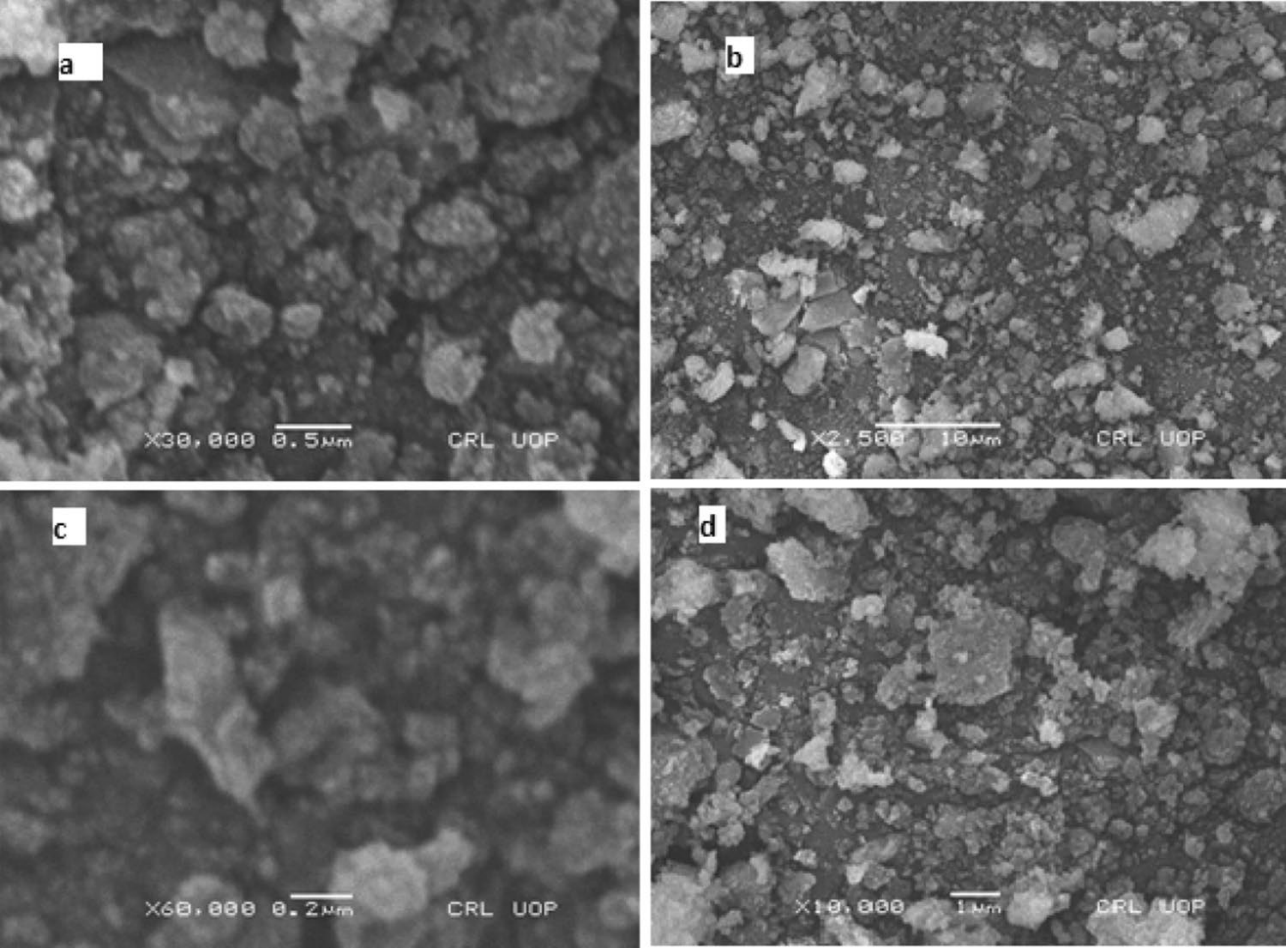
SEM of analysis of MgO (**a**), Ca–MgO (**b**) before degradation of thiamethoxam, MgO (**c**), Ca–MgO (**d**) after degradation of thiamethoxam.

To study the variation and changes in morphology and structure of MgO nanoparticles and Ca-doped MgO nanoparticles SEM analysis was executed before and after photocatalytic study. The images of MgO nanoparticles and Ca-doped MgO nanoparticles before and after degradation are shown in Fig. 6. The morphological changes brought about by the addition of Ca as a dopant metal can be seen in the image of Ca-doped MgO nanoparticles. It was found that the crystals of MgO nanoparticles and Ca-doped MgO nanoparticles consist of irregular shapes, clumps of very small crystals. The figure depicts the anatase of undoped MgO nanoparticles generate without calcium ions. It was also demonstrated in the figure that the crystal of MgO nanoparticles and Ca-doped MgO nanoparticles contain sub-micro-sized particles. The photocatalytic properties are related to the morphology, geometry, and particle size of the nanoparticles and their composite materials^40^.

#### EDX analysis

**Figure 7:**
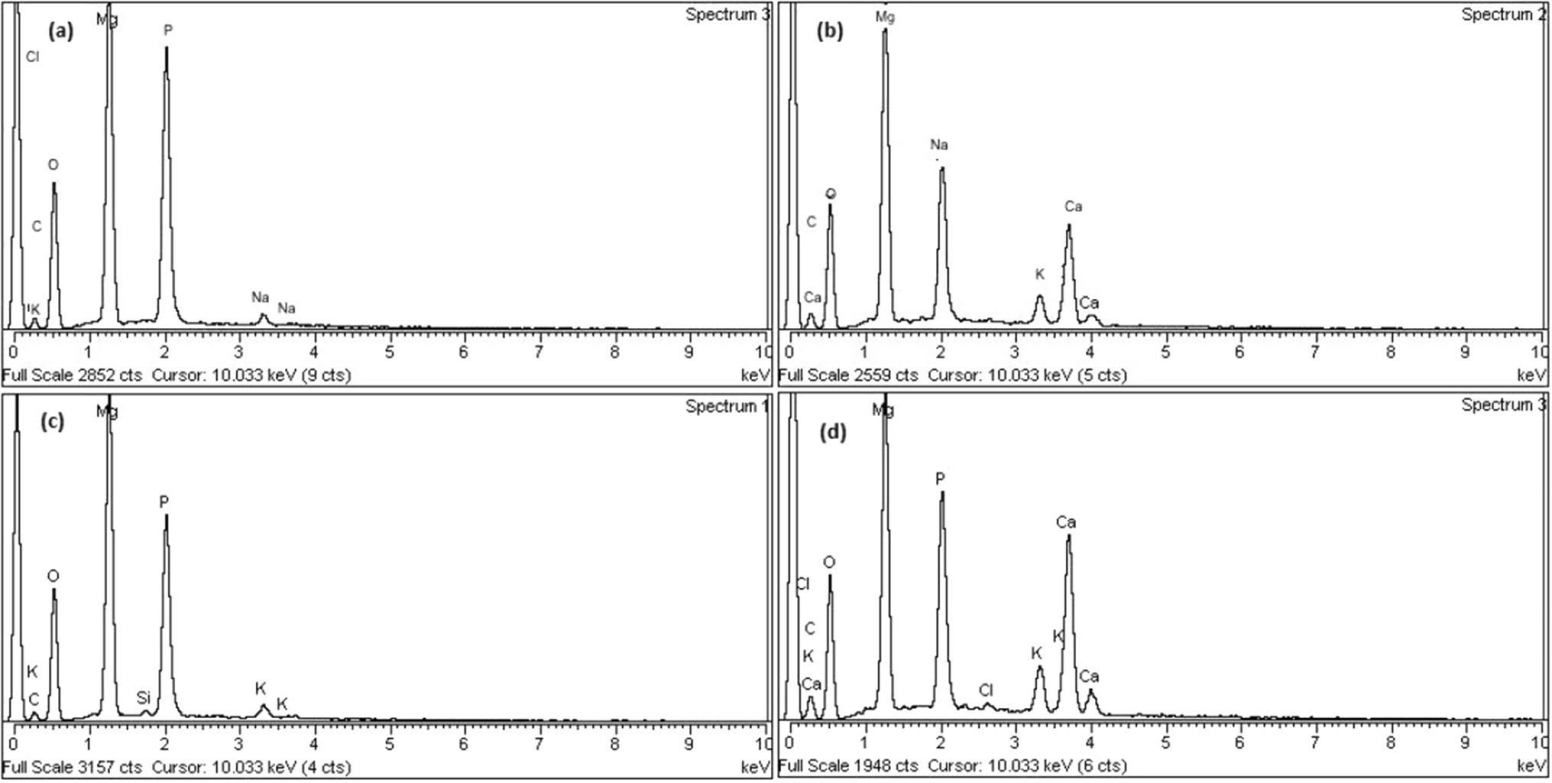
Elemental analysis of MgO and Ca-doped MgO before and after degradation of thiamethoxam.

To study the variation of MgO nanoparticles and Ca-doped MgO nanoparticles at elemental levels EDX analysis was performed. It can be seen from Fig. 7 that MgO nanoparticles and Ca-doped MgO nanoparticles before degradation contain C, O, Mg, and Ca having percentage composition (15.25%, 35.16%, 27.60%) and (29.76%, 25.62%, 28.65%, 9.97%) respectively. Moreover, an appropriate quantity of Na (18.34%) and Cl (4.00%) were also found in the crystalline structures of MgO nanoparticles and Ca-doped MgO nanoparticles. Whereas after degradation of MgO nanoparticles and Ca-doped MgO nanoparticles, the weight percentage of major constituents of C, O, Mg, and Ca were found to be (9.13%, 41.93%, 26.85%) and (14.58%, 38.2%, 16.92%, 8.62%) respectively. It has also been seen in the figure that P, K, and Si were present in the percent weight of 20.11%, 1.44%, and 0.55 respectively^44^. It may be inferred from the results that calcium atoms have successfully doped in the crystalline structure of MgO nanoparticles and the elemental composition of these catalysts was changed after degradation which indicates the photocatalytic degradation of thiamethoxam.

#### FT-IR analysis

**Figure 8:**
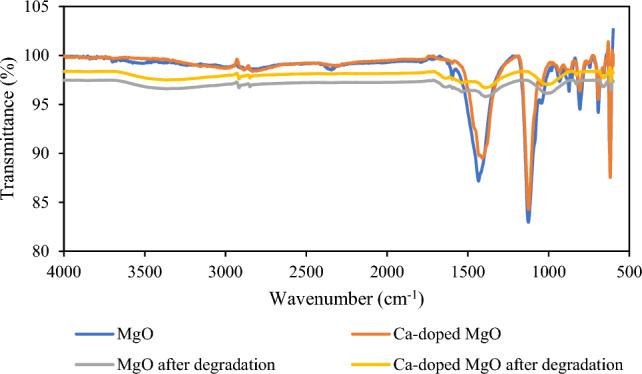
FTIR analysis of MgO nanoparticles and Ca-doped MgO nanoparticles.

FT-IR analysis was used to investigate the functional groups of MgO nanoparticles and Ca-doped MgO nanoparticles in the range of 4000–500 cm^−1^ and the results are shown in Fig. 8. Due to the reaction between MgO nanoparticles and water vapors, the Ca-doped MgO nanoparticles’ transmittance peak around corresponded to the typical stretching vibrations of the OH group. The peak intensity becomes reduced due to the presence of calcium as a dopant metal in Ca-doped MgO nanoparticles. The asymmetric stretching vibrations of carbonate ions and bending vibrational peaks were also observed. It has already been discussed that the decrease in peak intensity is due to the presence of Ca-doped MgO nanoparticles. The presence of MgO bending vibrations was also noticed in the figure^45^. The figure indicates the FTIR of MgO nanoparticles and Ca-doped MgO nanoparticles having characteristic peaks at 617, 693, 807, 1435, 619, 693, 809, 1125, and 1404 cm^−1^. The peaks around 1435 and 1404 cm^−1^ indicate the common band of the O–H group stretching mode^46^. The band observed at 1117 and 1125 cm^−1^ related to the C=O stretching mode^47^. The band shown at 624 cm^−1^ may be attributed to the Mg–O bond while the peak at 809 cm^−1^ was assigned for the pure MgO stretching. The decrease in peak intensity confirms the presence of doping of calcium atoms onto MgO^48^.

#### XRD

**Figure 9:**
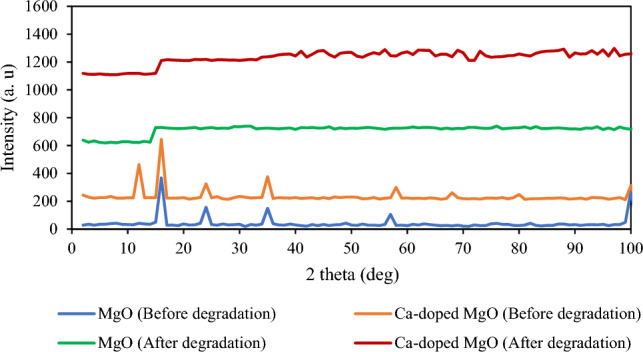
XRD analysis of MgO nanoparticles and Ca-doped MgO nanoparticles before and after degradation of thiamethoxam.

The XRD analysis was performed to comprehend the crystalline plane of MgO nanoparticles and Ca-doped MgO nanoparticles. The XRD pattern with 2 theta values shows characteristic peaks at 38°, 48°, and 53° as depicted in Fig. 9. This result indicates the plane of MgO nanoparticles which confirms that MgO has a cubic structure. These peaks are observed in both the MgO nanoparticles and Ca-doped MgO nanoparticles. However, a new peak appeared after the doping of calcium atoms in the crystalline structure of MgO nanoparticles. This result suggests that calcium atoms have been successfully doped in the crystalline structure of MgO nanoparticles^49^. Moreover, it may also have been seen in the figure that most of the peaks disappeared after photocatalytic degradation in both cases confirming the degradation of thiamethoxam. The mean crystalline size of MgO and Ca-doped MgO nanoparticles were evaluated using the Scherrer equation given below:

$$D = \frac{K\lambda }{{\beta COS\theta }}$$

where D is the mean crystalline size, K is the constant and has a value of 0.89, λ is the wavelength of X-rays in angstroms (0.154 Å), θ is the peak angle and β is the width at half maximum (FHWM) of the respective XRD peak.

The mean crystalline size of MgO and Ca-doped MgO nanoparticles were computed using the Origin software and were found to be 33 and 34 nm respectively.

### Stability and reusability study

**Figure 10:**
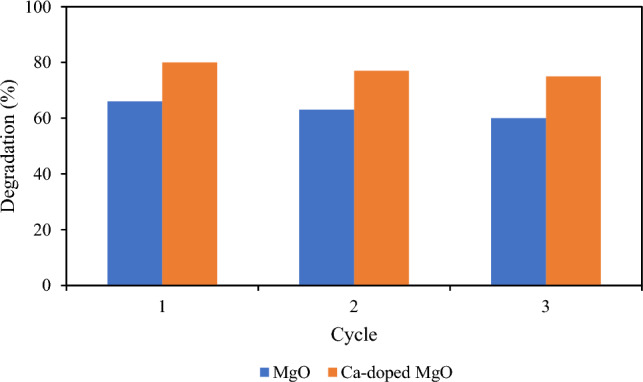
Stability and reusability study of MgO and Ca-doped MgO nanoparticles.

The same photocatalytic procedure was performed for the stability and reusability of experiments using the recovered photocatalysts. The nanoparticles of MgO and Ca-doped MgO were collected from the suspension by filtration and died at in oven at 80 °C for 2 h. The dried MgO and Ca-doped MgO nanoparticles were re-dispersed in another new thiamethoxam pesticide solution. The reusability of the photocatalysts was analyzed three times and the results are illustrated in the Fig. 10. It can be seen from the figure that degradation efficiency of both photocatalysts were decreased corresponding to more cycles of the photodegradation process. However, the decrease in degradation performance of MgO and Ca-doped MgO nanoparticles is marginally from 66 to 60% and 80–75% after 3 cycles respectively.

### UV–Vis spectrum of thiamethoxam

**Figure 11:**
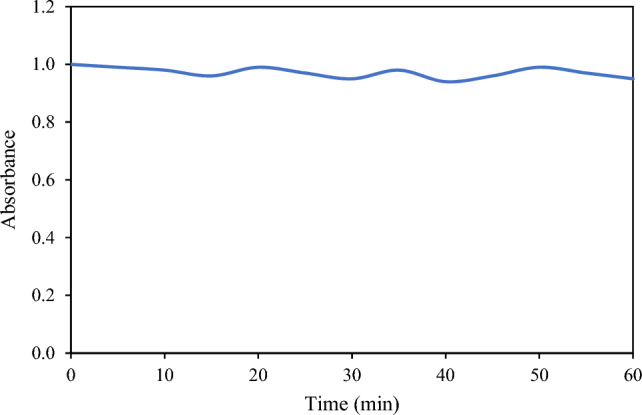
UV–Vis spectrum of thiamethoxam with respect to time.

The UV–Vis spectrum of standard solution of thiamethoxam (2 ppm) was recorded at its maximum wavelength of 240 nm and the result is shown in Fig. 11. The figure demonstrates that no appreciable change in the absorbance of thiamethoxam with respect to time was observed up to one hour.

### Comparative photocatalytic study

The photocatalytic performance of MgO and Ca-doped MgO nanoparticles was compared with other frequently used photocatalysts used for the degradation of thiamethoxam reported in the literature and presented in the Table 1. It can be obviously demonstrated from the table that photocatalytic performance of the synthesized materials is almost comparable with that of the materials reported in the literature.

**Table 1:** Comparison of photocatalytic performance of MgO and Ca-doped MgO against thiamethoxam with other photocatalysts reported in literature.

Photocatalyst	Degradation (%)	References
ZnO	77	^50^
TiO_2_	90	^51^
MgO	66	In present study
Ca-doped MgO	80	In present study

#### Kinetic study

**Figure 12:**
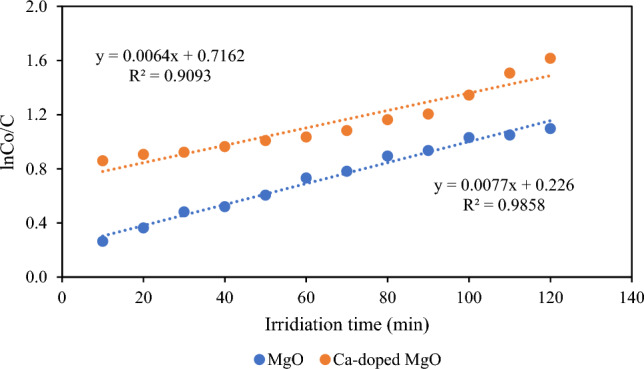
Plot of lnC_o_/C against irradiation time for kinetic study.

The most commonly used kinetic model; Langmuir–Hinshelwood was used to investigate the fitness of degradation data of thiamethoxam which is represented in the following equation:

$$ln\frac{{C_{o} }}{C} = k_{1} t$$

where C_o_ is the initial concentration of thiamethoxam (µgmL^−1^) and C is the final concentration of thiamethoxam (µgmL^−1^) after degradation at t time. Moreover, k_1_ (min^−1^) is rate constant and was evaluated from the slope of plot ln(C_o_/C) against irradiation time as depicted in Fig. 12. The values of the rate constant were found to be 7.7 × 10^–3^ min^−1^ and 6.4 × 10^–3^ min^−1^ for MgO and Ca-doped MgO nanoparticles respectively. The findings of kinetic study suggest that photocatalytic performance of MgO and Ca-doped MgO are comparable with each other, and similar result has been cited in the literature^52^.

#### Bandgap energy

**Figure 13:**
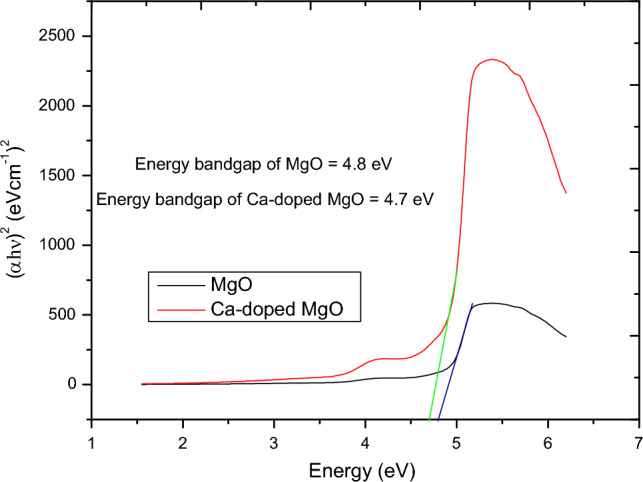
Calculation of bandgap energy of MgO and Ca-doped MgO nanoparticles.

The shifting of bandgap energy toward visible region improves the photocatalytic performance of the catalyst by decreasing the gap between valance band and conduction band^32^. Therefore, the bandgap energy of MgO and Ca-doped MgO nanoparticles has been calculated by plotting (αhυ)^2^ against photon energy (hυ) in the Origin software using the UV–Visible spectra of MgO and Ca-doped MgO nanoparticles. It can be illustrated from the Fig. 13 that bandgap energy of MgO and Ca-doped MgO nanoparticles was found to be 4.8 and 4.7 eV respectively. The result suggests that bandgap energy of the material was decreased slightly with doping of the calcium atoms in the crystalline structure of MgO nanoparticles.

## Conclusions

In this study, magnesium oxide (MgO) nanoparticles and calcium-doped magnesium oxide (Ca-doped MgO) nanoparticles were effectively synthesized their degradation activities were evaluated for thiamethoxam pesticide in aqueous media. The Characterization techniques such as XRD, SEM, EDX, and FT-IR analysis implied the confirmation of synthesis of MgO nanoparticles and Ca-doped MgO nanoparticles. The pH study indicates that maximum degradation of thiamethoxam pesticides was achieved at pH 5 in the case of MgO nanoparticles while in the case of Ca-doped MgO nanoparticles, maximum degradation was obtained at pH 8.0. The percentage degradation of thiamethoxam was initially increased with an increase in the dose of catalyst but decreased at the higher dose of the catalysts. The degradation of the pesticide was observed to be increased with an increase in contact time but decreased with a temperature rise. The degradation of pesticides increases at low initial concentrations but declined at higher concentrations. This study reveals that doping of MgO nanoparticles with calcium enhanced the degradation of thiamethoxam pesticide in aqueous media.

## Data Availability

Data is available under reasonable request to the corresponding author.

## References

[1] Rehman A (2015). Economic perspectives of major field crops oh Pakistan: An empirical study. Pac. Sci. Rev. B Hum. Soc. Sci..

[2] Aslam M (2016). Agricultural productivity current scenario, constraints and future prospects in Pakistan: A Review. Sarhad J. Agric..

[3] Sharma A (2019). Worldwide pesticide usage and its impact on ecosystem. SN Appl. Sci..

[4] Aktar MW, Sengupta D, Chowdhury A (2009). Impact of pesticides use in agriculture: Their benefits and hazards. Interdiscip. Toxicol..

[5] Muel R, Sabella G, Robba L, Manachini B (2017). Systematic review of the effects of chemical insecticides on four common butterfly families. Front. Environ. Sci..

[6] Maiehfisch P (2001). Chemistry and biology of thiamethoxam a second-generation neonicotinoid. Pest Manag. Sci..

[7] Malhotra N (2021). Physiological effect of neonicotinoid pesticides on non-targete aguatic animals—An updated review. Int. J. Mol. Sci..

[8] Nauen R, Ebbinghaus-Kintscher U, Salgado VL, Kaussmann M (2003). Thiamethoxam is a neonicotinoid precursor converted to clothianidin in insects and plants. Pestic. Biochem. Phys..

[9] Toolabi A (2017). Optimization of photochemical decomposition acetamiprid pesticide from aqueous solutions and effluent toxicity assessment by Pseudomonas aeruginosa BCRC using response surface methodology. AMB Expr..

[10] Alkayal NS, Hussein MA (2019). Photocatalytic degradation of atrazine under visible light using novel Ag@Mg_4_Ta_2_O_9_ nanocomposites. Sci. Rep..

[11] Kaur R, Kaur H (2021). Solar driven photocatalysis—An efficient method for removal of pesticides from water and wastewater. Biointerface Res. Appl. Chem..

[12] Lu Y, Li MC, Lee J, Liu C, Mei C (2023). Microplastic remediation technologies in water and wastewater treatment processes: Current status and future perspectives: Review. Sci. Total Environ..

[13] Bruckmann FS (2022). Adsorption and photocatalytic degradation of pesticides into nanocomposites: A review. Molecules.

[14] Arfaeinia H, Khaghani R, Fazlzadeh M, Poureshgh Y (2020). Silica-functionalized graphene oxide/ZnO as a photocatalyst for degradation of pirimphos-methyl from aqueous solutions. Desalin. Water Treat..

[15] Miguel N, Ormad M, Mosteo R, Ovelleiro JL (2012). Photocatalytic degradation of pesticide in natural water: Effect of hydrogen peroxide. Int. J. Photoenergy.

[16] Zandsalimi Y (2022). Photocatlytic removal of 2, 4-dichlorophenoxyacetic acid from aqueous solution using tungsten oxide doped zinc oxide nanoparticles immobilized on glass beads. Environ. Technol..

[17] Ansari SA (2023). Emerging NiO–rGO nanohybrids for antibiotic pollutant degradation under visible-light irradiation. Surf. Interfaces.

[18] Ansari SA (2023). Elemental semiconductor red phosphorus/ZnO nanohybrids as high performance photocatalysts. Ceram. Int..

[19] Jones BMF (2021). Simple fabrication and unprecedented visible light response of NiNb_2_O_6_/RGO heterojunctions for the degradation of emerging pollutants in water. New J. Chem..

[20] Ansari MZ, Ansari SA, Parveen N, Cho MH, Song T (2018). Lithium ion storage ability, supercapacitor electrode performance, and photocatalytic performance of tungsten disulfide nanosheets. New J. Chem..

[21] Ansari SA, Ansari SG, Foaud H, Cho MH (2017). Facile and sustainable synthesis of carbon-doped ZnO nanostructures towards the superior visible light photocatalytic performance. New J. Chem..

[22] Kumar A, Pandey G (2017). A review on the factors affecting the photocatalytic degradation of hazardous materials. Mater. Sci. Eng. Int. J..

[23] Zhu Q, Oganov AR, Lyakhov AO (2013). Novel stable compound in the MgO system under high pressure. Phys. Chem. Chem. Phys..

[24] Zheng Y (2019). Microscale flower-like magnesium oxide for highly efficient photocatlytic degradation of organic dyes in aqueous solution. RSC Adv..

[25] Allawi F, Juda AM, Radhi SW (2020). Photocatalytic degradation of methylene blue over MgO/*α*-Fe_2_O_3_ nanocomposite prepared by hydrothermal method. AIP Conf. Proc..

[26] Kumar A (2021). Construction of dual Z-scheme g C_3_N_4_/Bi_4_Ti_3_O_12_/Bi_4_O_5_I_2_ heterojunction for visible and solar powered coupled photocatalytic antibiotic degradation and hydrogen production: Boosting via I^−^/I_3_^−^ and Bi^3+^/Bi^5+^ redox mediators. App. Catal. B Environ..

[27] Gul I (2020). Solar light responsive bismuth doped titania with Ti^3+^ for efficient photocatalytic degradation of flumequine: Synergistic role of peroxymonosulfate. Chem. Eng. J..

[28] Sayed M (2018). Narrowing the band gap of TiO_2_ by co-doping with Mn^2+^ and Co^2+^ for efficient photocatalytic degradation of enoxacin and its additional peroxidase like activity: A mechanistic approach. J. Mol. Liq..

[29] Iqbal J (2022). Visible light driven doped CeO_2_ for the treatment of pharmaceuticals in wastewater: A review. J. Water Process. Eng..

[30] Iqbal J (2020). Synthesis of nitrogen-doped Ceria nanoparticles in deep eutectic solvent for the degradation of sulfamethaxazole under solar irradiation and additional antibacterial activities. Chem. Eng. J..

[31] Iqbal J (2022). Efficient removal of norfloxacin using nano zerovalent cerium composite biochar-catalyzed peroxydisulfate. J. Clean. Prod..

[32] Khan JA (2023). Synthesis of N-doped TiO_2_ nanoparticles with enhanced photocatalytic activity for 2,4-dichlorophenol degradation and H_2_ production. J. Environ. Chem. Eng..

[33] Shah NS (2022). Enhanced solar light photocatalytic performance of Fe–ZnO in the presence of H_2_O_2_, S_2_O_8_^2−^, and HSO_5_^−^ for degradation of chlorpyrifos from agricultural wastes: Toxicities investigation. Chemosphere.

[34] Vargas XM, Marin JM, Restrepo G (2015). Characterization and photocatalytic evaluation (UV–Visible) of Fe-doped TiO_2_ systems calcined at different temperatures. J. Adv. Oxid. Technol..

[35] Danish MSS (2021). Photocatalytic applications of metal oxides for sustainable environmental remediation. Metals.

[36] Nair MG, Nirmala M, Rekha K, Anukaliani A (2011). Structural, optical, photo catalytic and antibacterial activity of ZnO and Co doped ZnO nanoparticles. Mater. Lett..

[37] Mulpuri RK, Tirukkovalluri SR, Imandi MR, Alim SA, Kapuganti VDL (2019). Zinc and boron co-doped nanotitinia with enhanced photocatalytic degradation of acid red 6A under visible light irradiation. Sustain. Environ. Res..

[38] Adeel M, Saeed M, Khan I, Muneer M, Akram N (2021). Synthesis and characterization of Co–ZnO and evaluation of its photocatalytic activity for the degradation of methyl orange. ACS Omega.

[39] Dehghani MH, Fadaei AM (2012). Photocatalytic oxidation of organophosphorus pesticide using zinc oxide. Res. J. Chem. Environ..

[40] Rao TS, Segne TA, Susmitha T, Kiran AB, Subrahmanyam C (2012). Photocatalytic degradation of dichlorvos in visible light by Mg^+2^–TiO_2_ nanocatalyst. J. Photocatal. Mater..

[41] Krishnasamy L, Krishna K, Subpiramaniyam S (2022). Photocatalytic degradation of atrazine in aqueous solution using La-doped ZnO/PAN nanofibers. Environ. Sci. Pollut. Res. Int..

[42] Tabassum A (2020). Degradation of acetamiprid using graphene-oxide-based metal (Mn and Ni) ferrites as Fenton like photocatalysts. Water Sci. Technol..

[43] Molla MAI (2018). Photocatalytic degradation of fenitrothion in water with TiO_2_ under solar irradiation. Water Conserv. Manag..

[44] Haq AU (2018). Removal of butachlor from aqueous solution using cantaloupe shell powder: Kinetic, equilibrium and thermodynamic studies. Int. J. Environ. Sci. Technol..

[45] Ikram M (2021). Graphene oxide-doped MgO nanostructures for highly efficient dye degradation and bactericidal action. Nanoscale Res. Lett..

[46] Raghavendra M, Lalithamba HS, Sharath BS, Rajanaika H (2017). Synthesis of N-protected formamidesfrom amino acids using MgO nanocatalyst: Study of molecular docking and antibacterial activity. Sci. Iran..

[47] Kandiban, M., Vigneshwaran, P., & Potheher, I. V. Synthesis and characterization of MgO nanoparticles for photocatalytic applications. In *Conference: National Conference on Advances in Crystal Growth and Nanotechnology At: Kottayam, Kerala* (2015).

[48] Jawwad MAS, Murti RHA, Wang Y-F, You S-J (2020). FTIR analysis of MgO/TiO_2_ nanocomposite on adsorption of remazol turquoise blue dye. Nusant. Sci. Technol. Proc..

[49] Patil HR, Murthy ZVP (2016). Vanadium-doped magnesium oxide nanoparticles formation in presence of ionic liquid and their use in photocatalytic degradation of methylene blue. Acta Metall. Sin. (Engl. Lett.).

[50] Banic N (2016). Efficiency of neonicotinoids photocatalytic degradation by using annular slurry reactor. J. Chem. Eng..

[51] Zabar R, Komel T, Fabjan J, Kralj MB, Trebse P (2012). Photocatalytic degradation with immobilised TiO_2_ of three selected neonicotinoid insecticides: Imidacloprid, thiamethoxam and clothianidin. Chemosphere.

[52] Khalid H, Haq AU, Naqvi SAR, Usman M, Bokhari TH (2023). Enhancement of photocatalytic activity of Ba-doped CoO for degradation of Emamectin benzoate in aqueous solution. Environ. Monit. Assess..

